# Evaluating the relationship between Clinical G6PD enzyme activity and gene variants

**DOI:** 10.7717/peerj.16554

**Published:** 2024-01-03

**Authors:** Xinyi Zhou, Zheng Qiang, Sufen Zhang, Yuqiu Zhou, Qizhi Xiao, Gongjun Tan

**Affiliations:** 1 Department of Clinical Laboratory & Zhuhai Institute of Medical Genetics, Zhuhai Maternity and Child Healthcare Hospital, Zhuhai, Guangdong, China; 2 Pathology Department, Zhuhai Maternity and Child Healthcare Hospital, Zhuhai, China; 3 Department of Clinical Laboratory & Zhuhai Institute of Medical Genetics, Zhuhai Maternity and Child Healthcare Hospital, Guangdong, China

**Keywords:** G6PD, Enzyme activities, Genetic variants

## Abstract

Glucose-6-phosphate dehydrogenase (G6PD) is a the first and rate-limiting enzyme that plays a critical role in G6PD deficiency, the most common enzyme disorder worldwide, is related to intravascular hemolysis. To determine the clinical enzyme activity level in different G6PD variants, we evaluated 15 variant from 424 clinical blood samples by using multicolor melting curve analysis and DNA sequencing. The results showed that the enzyme activities of the hemizygous deficient were 1.5–2.4 U/gHb, which was significantly lower than those of the heterozygous (*P* < 0.001) and the compound heterozygous variants (*P* < 0.05). Since the hemizygous of c.1024C > T (Chinese-5) mutation affects the kinetic parameters of G6PD and increase utilization of analogues, its enzyme activity is more than those of other mutations that mutated in the β+α region of G6PD. The heterozygous enzyme levels ranged from 6.5–20.1 U/gHb; and there was no significant difference among different heterozygous variants (*P* > 0.05). The enzyme activity levels of the compound heterozygous mutation were mainly in the range of 1.7–3.8 U/gHb, which was much lower than that of the heterozygous mutation (*P* < 0.001). In summary, our findings revealed that the enzyme activity of G6PD in blood have a significant relationship with genotype of G6PD.

## Introduction

Glucose-6-phosphate dehydrogenase deficiency in the red blood cell can cause acute hemolytic anemia when exposed to oxidative agents such as drugs, certain food or infections (Wu, Zhu & Zhong, 2020). It leads to reduction of reduced nicotinamide adenine dinucleotide phosphate (NADPH), which impairs downstream gluthathione cycle (GSH), leading to less resistance to oxidative damage and causes red cell hemolysis (Saul, Jaime & American, 2016). Patients with severe G6PD deficiency can undergo acute hemolysis that can greatly endangers their lives, due to oxidative stress induced by intake of certain foods such as fava beans or oxidative drugs and certain chemicals. These cases are often found in the provinces of southern China, like Guangdong, Guangxi, Hainan, Yunnan, Guizhou, and certain other cities or areas. Naylor et al. (1996) constructed a G6PD three-dimensional space model structure and found that the G6PD dimer is composed of two identical subunits, with each consisting of two domains: the substrate domain and the β+α structure. The substrate domain is responsible for the binding to the substrate, while the β+α structure is related to the polymerization of subunits. Enzyme activity occurs only in dimeric or tetrameric forms (Pes & Dore, 2022). G6PD deficiency is an X-linked hereditary defect caused by mutations in the *G6PD* gene (Lin, Yang & Ye, 2016). So far, over 240 variants of *G6PD* gene (https://databases.lovd.nl/shared/variants/G6PD/unique) have been defined in relation to G6PD deficiency cases worldwide. Over 35 G6PD variants have been discovered in the Chinese population, which are single base mutations. In these mutations, three mutation types are the most common and only detected in the Chinese population, namely, 1376G > T (Canton), 1388G > A (Kaiping) and 95A > G (Gaohe) (Zhen, Yan & Ning, 2014). However, there are currently few clinical studies on the relationship between G6PD gene variants and the G6PD enzyme activity. Therefore, to explore the correlation between G6PD enzyme activity and G6PD genotype, we collected G6PD enzyme quantification and G6PD gene mutation data from Zhuhai Maternal and Child Healthcare Hospital during 2017–2020 for statistical analysis.

## Materials and Methods

### Sample collection and research method

A total of 510 peripheral blood samples were collected from patients with suspected G6PD deficiency from the Prenatal Diagnosis and Neonatology Department at Zhuhai Maternal and Child Healthcare Hospital from 2017–2020, including 287 males and 223 females, aged from 16–38 years old (Table 1). Because this experiment is based on the data of clinical examination, the specimen for further analysis is the remaining specimen of the patient. This study was approved by the Institutional Ethics Committee of Zhuhai Maternal and Child Healthcare Hospital. Approval No: (20210921001).

**Table 1:** Clinical characteristics of patients.

	All	Male	Female
Number of subjects	510	277 (56%)	233 (44%)
Age			
<20 years old	278 (54.5%)	185	93
20–40 year old	230 (45.0%)	101	139
>40 year old	2 (0.39%)	1	1
Genotype			
Heterozygous	123 (24.11%)	0 (%)	123 (100%)
Hemizygous	277 (54.31%)	277 (100%)	0 (%)
Compound Heterozygous	110 (21.56%)	0 (%)	110 (100%)

### Enzyme activity measurement and genotype

G6PD activity reagents purchased from Guangzhou Kefang Biotechnology Co., Ltd., Guangzhou, China. The ratio between G6PD and 6PGD absorbance values were analyzed by using an automatic biochemical analyzer (Hitachi 7180 System; Hitachi HiTech, Tokyo, Japan). A testing result that of G6PD/6PGD < 1.0 and G6PD activity less than 25 U/g Hb was considered as G6PD deficiency; simultaneously, a female heterozygous G6PD sample test ratio within 1.0 ± 5% is considered suspicious, which means that the result is near the critical value. Meanwhile, the principle of multicolor melting curve analysis (MMCA) technology (Xiamen Zhishan Biotechnology Co., Ltd., Xiamen, China) was used to identify mutations in the blood samples. Specifically, two PCR amplification systems (A and B tubes) and four fluorescence channels (FAM, CY5, HEX, and ROX) were recorded according to the target sequence, and melting point changes of the hybridization probes were used to detect 16 common G6PD gene mutation sites (Xia, Chen & Tang, 2016). To further detect rare variants, primers were designed at the 2nd–13th exons and UTR region of the G6PD gene and Sanger sequencing was employed (Table 2). The primers were synthesized by Shanghai Bioengineering Technology Co., Ltd., China.

**Table 2:** G6PD gene of forward and reverse primers sequences.

Primer pairs	Upstream primer (5′–3′)	Downstream primer (5′–3′)
1	TGTACACCCTGAATGAAGGCTG	TGCAAGTGCATATGCACACCA
2	TTGAATCTCGGGGCTCTTCTG	GGTCTCAAGGAAGTACGAGAGCA
3	TCTGGATGTGCAGAGCTGCTAAG	TTTGCTTTACTACCCCCGCAT
4	AGGTGTTGAGCCAGAGGGTCAT	CTTTCCAGCCCGGTCTGATAG
5	AGTGATAGCATCACCATGTCCTTC	AGGCCTGGGACATGACAACTT
6	GGGCCTCAGCTTGTTCATCA	CCTCACCTGCCATAAATATAGGG
7	TCAGCAAGACACTCTCTCCCTCAC	TCCTCAGGGAAGCAAATGACAAG

### Statistical analysis

Data were analyzed using GraphPad Prism 5.0 software (GraphPad Software, La Jolla, CA, USA). All data were expressed as mean ± standard deviation. The mean comparison between groups was analyzed by one-way analysis of variance. *P*-value < 0.05 was considered statistically significant; *P < *0*.05, **P < 0.01, and ***P < 0.001.

## Results

### G6PD mutation genotypes

**Figure 1:**
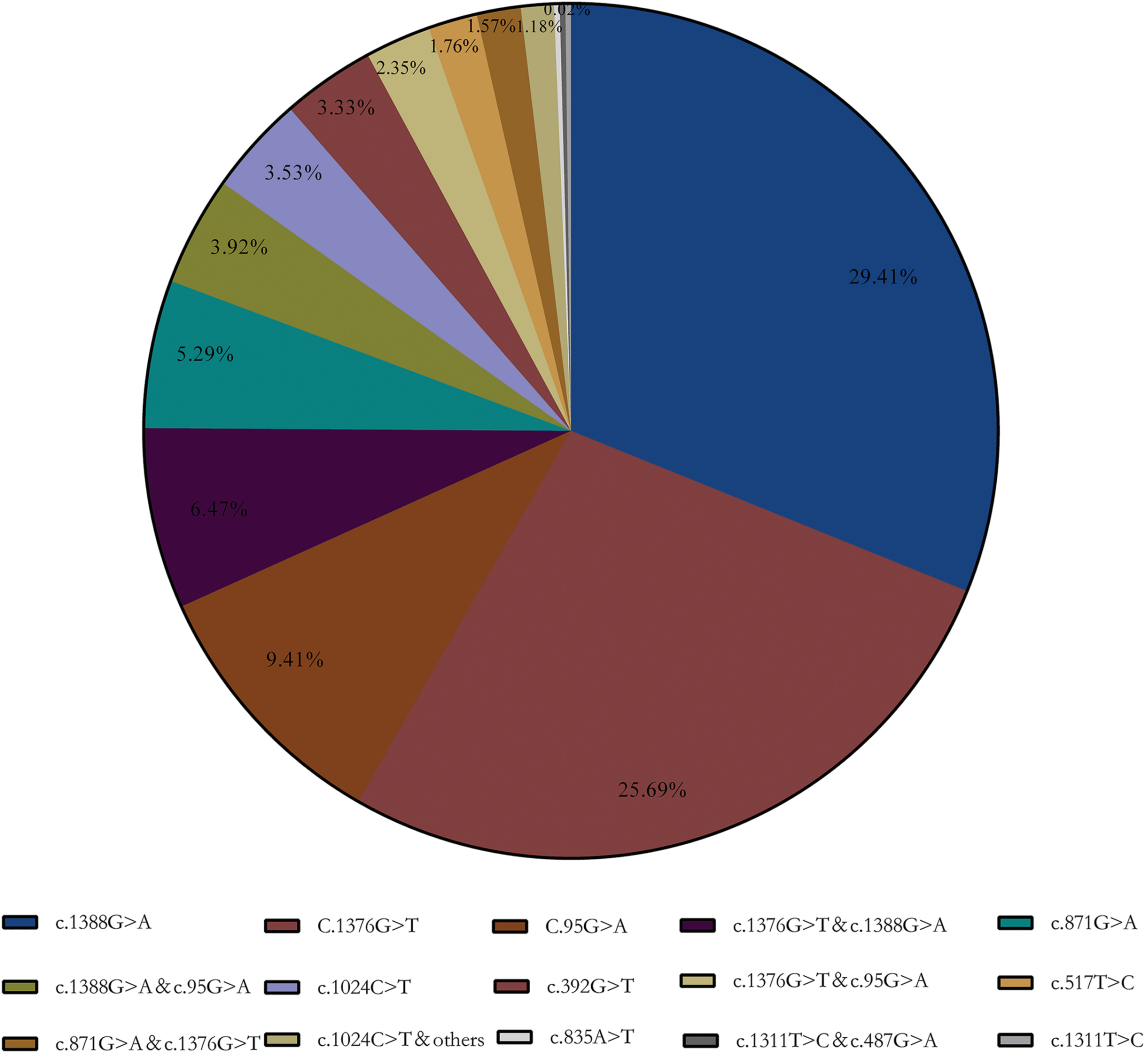
G6PD gene mutation and its composition ratio. c.1388G > A, c.1376G > T and c.95A > G are the most common types of G6PD gene mutations in the Chinese population, accounting for 64.51% (29.41%, 25.69% and 9.41%, respectively).

Enzymatic test results showed that 424 samples below the normal reference range (<25 U/g Hb), which included in subsequent statistics. The final genotyping result showed that a total of 123 heterozygous, 277 hemizygous deficient, and 112 compound heterozygous were included in our collected samples. A total of 15 G6PD gene mutation sites were detected using the MMCA method combined with Sanger sequencing. Furthermore, DNA sequencing revealed that three samples were unusual: a Chinese-1 type mutation (c.835A > T), a heterozygous mutation (c.1311T > C), and a compound mutation Mahidol (c.487G > A) and c.1311T > C. Meanwhile, the results showed that Kaiping (c.1388G > A), Canton (c.1376G > T), and Gaohe (c.95A > G) are the most common types of *G6PD* gene variants in the Chinese population, accounting for 64.51% (29.41%, 25.69%, and 9.41%, respectively). Among them, *G6PD* compound mutation accounted for 15.69% and c.1376G > T & c.1388G > A (6.47%) was the most common especially (Aa) (Fig. 1).

### Enzyme activity values of three types of variants

**Figure 2:**
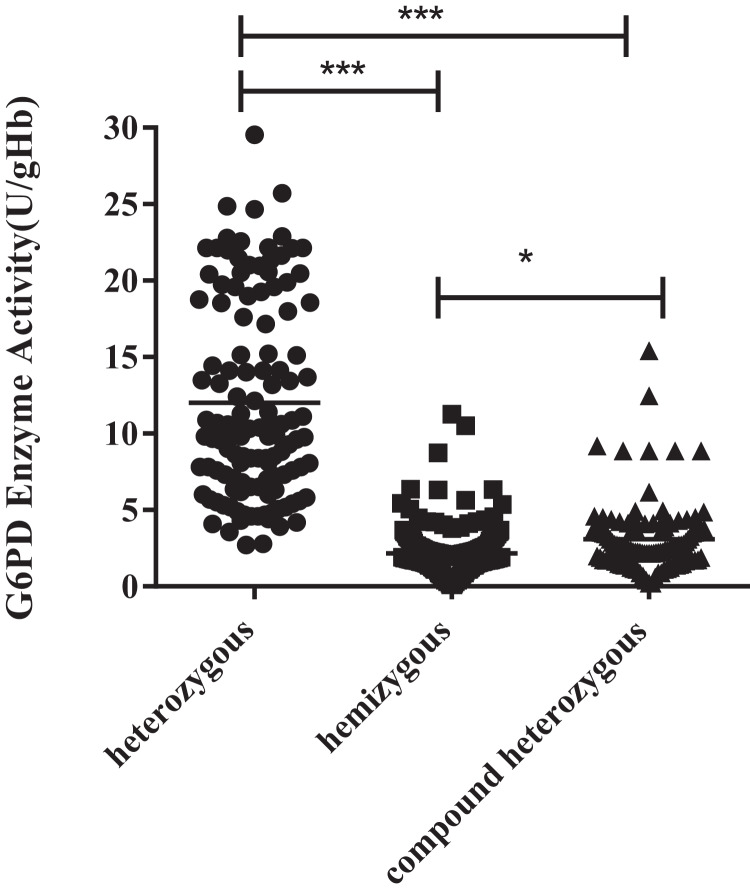
Enzyme activity values of three types mutations. The enzyme activity of the hemizygous mutations was significantly lower than that of the heterozygous (***p < 0.001) and compound heterozygous mutation (*p < 0.05). Furthermore, there was also a significant difference between compound heterozygous and heterozygous (***p < 0.001).

The enzyme activity of the hemizygous variants was significantly lower than the heterozygous (*P* < 0.001) and compound heterozygous variants (*P* < 0.05). Furthermore, there was also a significant difference between compound heterozygous and heterozygous (*P* < 0.001). It can be obtained from the figure that hemizygous deficient has similar G6PD activity as compound heterozygous (Fig. 2).

### Heterozygous mutation and G6PD enzyme activity

**Figure 3:**
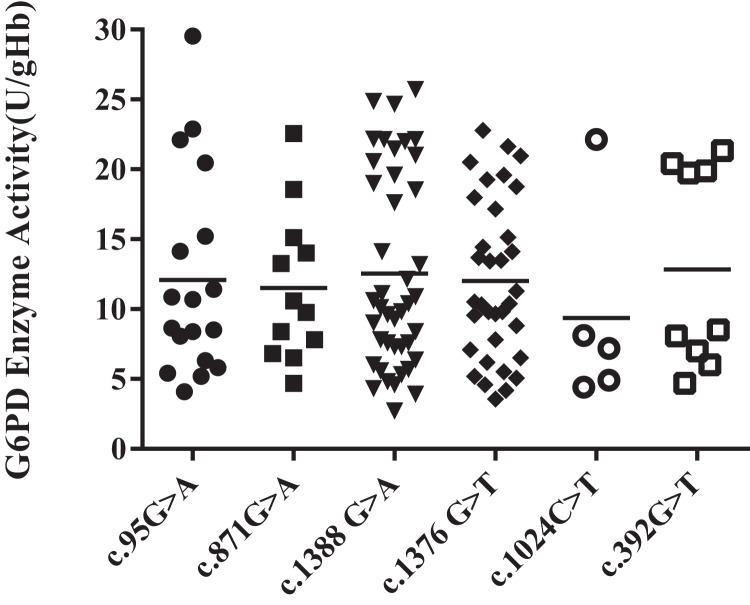
Distribution of enzyme activity of G6PD gene with heterozygous mutation. There is no difference among heterozygous mutations.

Among all the heterozygous mutation sites, Kaiping (c.1388G > A) was the most frequent mutation, accounting for 35%. The enzyme activity levels of the Viangchan (871G > A) mutant was more uniform, while that of Chinese-5 (c.1024C > T) mutant was less evenly distributed. Additionally, the enzyme activity values of Gaohe (c.95A > G), Kaiping (c.1388G > A) and QuingYuan (c.392G > T) mutant were more scattered. However, the difference in enzyme activity levels between each group was not obvious (*P* > 0.05) (Fig. 3).

### Hemizygous mutation and G6PD enzyme activity

**Figure 4:**
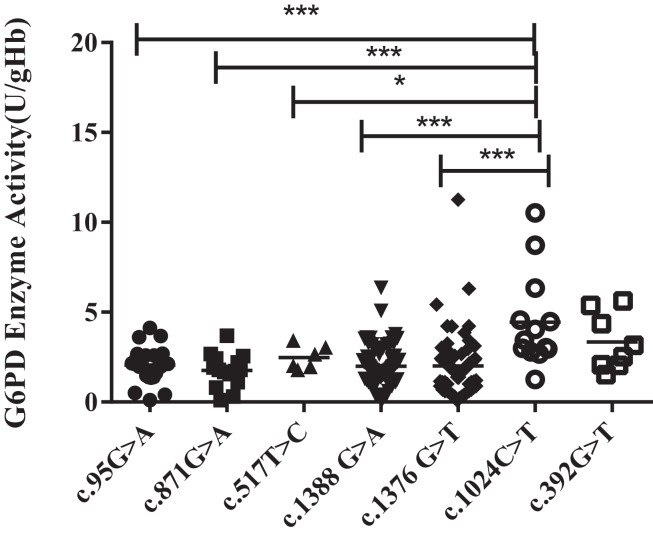
Distribution of enzyme activity of G6PD gene with hemizygous mutation. Enzyme activity of c.1024C > T was significantly higher than that of c.871G > A, c.95A > G, c.1388G > A and c.1376G > T (***P < 0.001), c.517T > C (*P < 0.05), expect c.392G > T *vs* c.1024C > T (*P* > 0.05).

Unlike the heterozygous G6PD gene variants (all females), enzyme activities of the hemizygous G6PD variants were approximately 1.56–2.46 U/g Hb, which was significantly lower than that of the heterozygous G6PD gene variants (*P* < 0.001). Kaiping (c.1388G > A) and Viangchan (c.871G > A) mutations were accounted for around 38.9% and 35% respectively, The enzyme activity of c.1024C > T mutant was significantly higher than that of mutant Viangchan (c.871G > A), Gaohe (c.95A > G), Kaiping (c.1388G > A), or Canton (c.1376 G > T) *(P* < *0.001)*. While compared with c.517T > C mutant, the enzyme activity value of Chinese-5 (c.1024C > T) was slightly higher (*P* < 0.05). The enzyme activities of Chinese-5 (c.1024C > T) and c.392G > T mutants were relatively scattered, which results in no statistical difference between them (*P* > 0.05) (Fig. 4).

### Compound heterozygous mutation and G6PD enzyme activity

**Figure 5:**
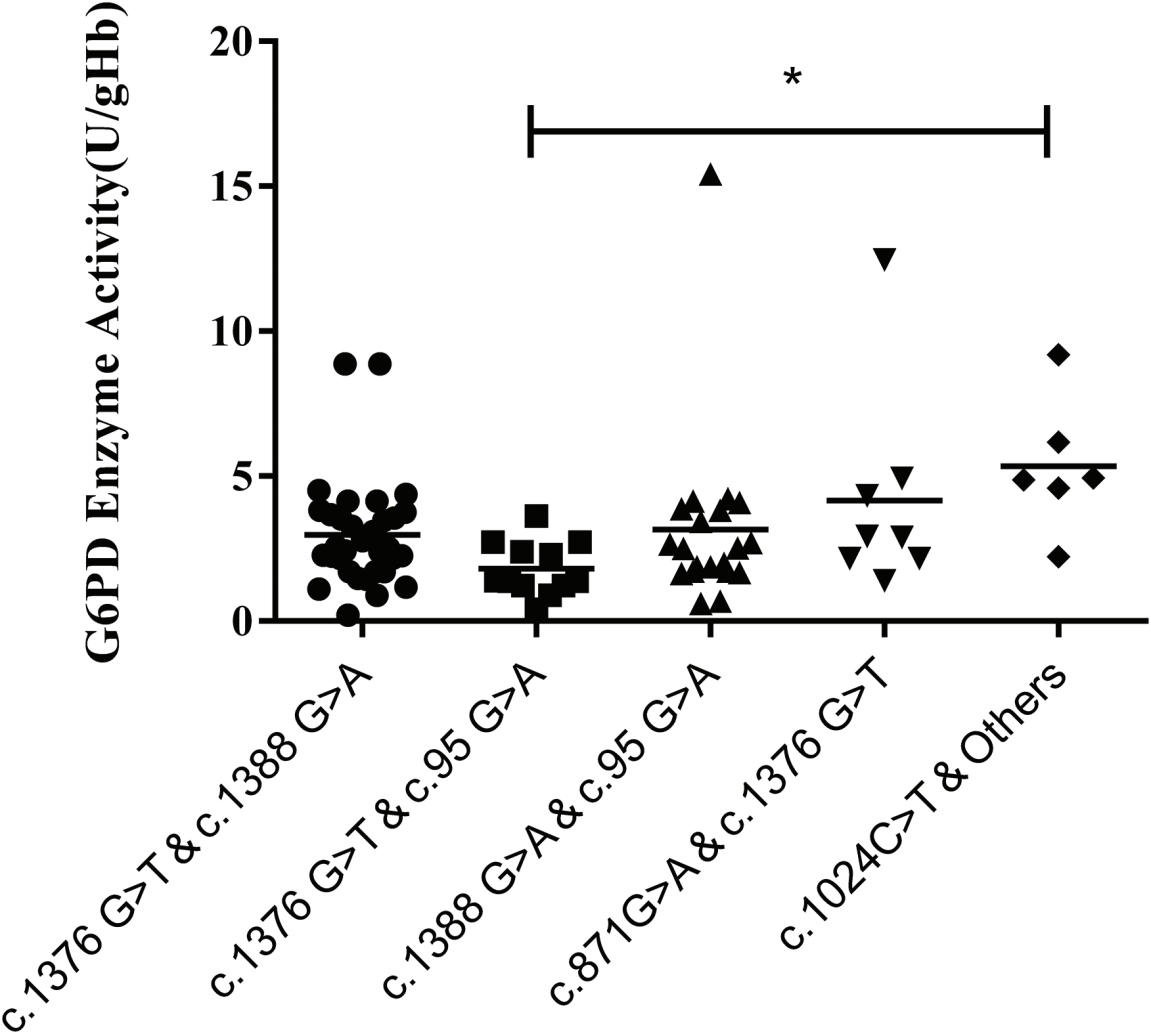
Distribution of enzyme activity of G6PD gene with compound heterozygous mutation. Enzyme activity of c.1024C > T & Others (4.0–6.9 U/gHb) was significantly higher than the c.1376G > T & c.95G > A (*P < 0.05).

Results of the compound heterozygous G6PD mutations showed that the enzyme activity of each mutant ranged from 1.7–3.8 U/g Hb, which was much lower than that of the heterozygous variants (*P* < 0.001). Meanwhile, among the compound heterozygous variants, c.1376G > T & C. 1388G > A was the most frequent mutations, accounting for 41.7% of the total. In addition, a significant difference in enzyme activity was seen between the compound heterozygous variants and the hemizygous variants *(P* < *0.05)*. While a significant difference was found between c.1376G > T & c.95G > A and c.1024C > T & other mutations (*P* < 0.05) (Fig. 5).

## Discussion

G6PD is a housekeeping enzyme found in all cells and tissues. It is also the initiation enzyme of pentose phosphate bypass metabolism, which can catalyze the conversion of glucose-6-phosphate to glucose-6-phosphogluconolactone and to produce the reduced NADPH. NADPH is not only a hydrogen donor synthesized by various substances in the body, but also is used to maintain glutathione in its reduced state, which is essential for the stability of red blood cell membranes and elimination of free radicals in the body. G6PD deficiency is more frequently found in the tropical and subtropical regions around the world where malaria was or is endemic (Xia, Chen & Tang, 2016). According to previous epidemiological studies in malaria endemic areas, G6PD deficiency and thalassemia can effectively reduce susceptibility to malaria (Alina et al., 2020). Under normal conditions, G6PD enzyme activity is relatively high in reticulocytes. However, once reticulocytes develop into mature red blood cells, G6PD enzyme protein synthesis is stopped, and its enzyme activity would exponentially decrease with the senescence of red blood cells (Harcke, Rizzolo & Harcke, 2019). Yang, Tai & Chu (2020) tested 410 cases of G6PD deficient newborns (7–90d) with genotyping, and found that the G6PD enzyme activity levels were significantly different between the female heterozygous and male hemizygous deficient groups. However, they neither clarify the specific G6PD gene variants nor the relationship between enzyme activities and different variants. In our study, we used a quantitative G6PD/6GPD ratio analysis method to conduct a preliminary screening for G6PD levels, and then employed MMCA combined with DNA sequencing to detect the type of G6PD gene mutation to determine the clinical G6PD enzyme activity level in different genotypes. The difference is especially obvious in the hemizygous. Due to the difference in the number of X chromosomes between females and males, carrying the same gene mutation had different effects on males and females. The G6PD hemizygous enzyme activity of males with G6PD mutation decreased significantly to about 90% of normal. At the same time, we found that the single nucleotide polymorphism (SNP) site c.1311T > C caused a synonymous variant. Interestingly, previous findings have reported that the c.1311T > C mutation on exon 11 of the G6PD gene is a synonymous mutation which was found in both normal and G6PD-deficient patients. It was also considered a crucial genetic marker on the X chromosome for X chromosome inactivation researches (Shu, Qing & Fang, 2015).

In 2000, Au, Gover & Lam (2000) used X-rays to determine the G6PD crystal model and found that G6P, NADP+ binding sites and the C-terminus of the G6PD peptide chain are important functional regions (Saha, Hong & Low, 1995). Three conserved regions were affirmed in the catalytic domain a nine-residue peptide (residues 196–206 in human enzyme) that is responsible for binding and catalysis of G6P, a nucleotide-binding fingerprint (residues 38–44) involved in binding the catalytic NADP+ coenzyme, and a 170–174 between the catalytic NADP+ and G6P substrate binding sites (Hwang, Mruk & Rahighi, 2018). Residues are significant for providing proper orientation of coenzyme or substrate through hydrogen binding or electrostatic interactions within each binding pocket. The depletion of those residues leads to a lower binding affinity to zymolytic or coenzyme, or an altered electrostatic interactions that reduces catalytic efficiency (Kotaka et al., 2005). Among the variants, Viangchan (c.871G > A), Kaiping (c.1388G > A), Canton (c.1376G > T) and Chinese-5 (c.1024C > T) are located in the β+α domain, and Gaohe (c.95G > A) Nankang (c.517T > C) and QuingYuan (c.392G > T) are found in the NADP+ binding domain. The peptide of 33–44 in the N-terminal are the second binding site, and the lysine at position 205 is the G6P binding site (Sirdah et al., 2021).

Most of the genes that cause serious defects in enzyme activity occur near the NADP+ binding domain, and those that are mutated near the carboxyl-terminal and amino-terminal regions may cause mild clinical symptoms. Prior research has pointed out that the Chinese-5 (c.1024C > T) mutation is located in the β-tetramer subunit binding region of exon 9, which is unnecessary for G6PD enzyme activity. According to SIFT and PolyPhen2 algorithm evaluations, the score of the severity of type III variants is only 0.5. Moreover, Saha et al. showed that Km values of the c.1024C > T mutation for G6P and NADP+ binding are 40 and 7 mol/L, respectively, which are higher than those of Canton (c.1376G > T) and Kaiping (c.1388G > A) (Au, Gover & Lam, 2000). Additionally, Chinese-5 (c.1024C > T) can also reduce G6PD enzyme consumption by increasing utilization of the 6-phosphate galactose and 2-deoxy-glucose-6 phosphate analogs, thereby allowing its enzyme activity comes to be higher than those of other point mutation types. Since females have two X chromosomes, one of the two G6PD alleles is in a random inactivation state. In consideration of the difference in mosaicism between enzyme-deficient red blood cells and normal red blood cells, the enzyme activity of female heterozygous G6PD deficient patients can have large heterogeneity, which can be significantly reduced, or it can be in the normal range, which results in various clinical manifestations.

Through this study we found that the enzyme activity of heterozygous and compound heterozygous G6PD mutants are not distinguished depending on the gene mutation types. Among the hemizygous variants types, the enzyme activity of Chinese-5 (c.1024C > T) at the G6PD mutation site in exon 9 was significantly higher than that of other mutation types. This finding promoted us to speculate that the Chinese-5 (c.1024C > T) mutation site may affect the kinetic parameters of the G6PD enzyme and increase utilization of analogues, such as galactose 6-phosphate and 2-deoxy-glucose-6 phosphate, moving it toward a single step to reduce G6PD enzyme consumption so that its enzyme activity can be higher than those of other mutation sites. However, in the present study, its specific mechanism was not unveiled, and more data needs to be collected for further exploration.

## Supplemental Information

10.7717/peerj.16554/supp-1 Supplemental Information 1 Raw data.

## Abbreviations

G6PD: glucose-6-phosphatedehy dehydrogenase
GSH: glutathione
NADPH: nicotinamide adenine dinucleotide phosphate
UTR: untranslated regions
MMCA: multicolor melting curve analysis
RDB: reverse dot blot assay
SNP: single nucleotide polymorphism

## Additional Information and Declarations

### Data Availability

The following information was supplied regarding data availability: The raw data is available in the Supplemental File.
